# Supplementary material for: Tumoral switch in NUMB splicing changes essential transcription pathways and induces malignant properties in tumour cells
Source: Genome Biol. 2026 Jun 24;27:285. doi: 10.1186/s13059-026-04170-3 (PMC13551979; doi:10.1186/s13059-026-04170-3)
Supplement: Supplementary file 1 [file 13059_2026_4170_MOESM1_ESM.docx]

**Fig S1. Overview of the study workflow and computational analyses.** The diagram summarizes the sequential datasets and analysis steps used throughout the study, from pan-cancer TCGA alternative splicing profiling and the definition of a NUMB isoform framework (exon 3/exon 9 inclusion and NUMBL), to TCGA-BRCA paired tumour/non-tumour analyses defining the NUMB isoform switch signature and associated transcriptional programs. NUMB isoform groups are linked to gene networks and functional pathways, and isoform dynamics are evaluated across passages in patient-derived xenograft (PDX) samples. A NUMB-score based on isoform balance is then defined in CCLE-BRCA, used to stratify cell lines into functionally distinct states, and associated with pathway programs and drug response patterns (GDSC). Finally, the NUMB-score is clinically validated in the full TCGA-BRCA cohort, including associations with tumour status, AJCC stage, molecular subtypes and overall survival (KM/OS), supporting its clinical relevance as a prognostic marker.

**Fig S2**. **NUMB expression behaves as a marker of poorer or better survival in breast cancer.** Kaplan–Meier plots from independent breast cancer cohorts show that lower NUMB expression is associated with better survival in GSE25066, GSE162228, GSE20685 and GSE1456, whereas it is associated with worse survival in GSE42568 and GSE12093.

**Fig S3**. **Histograms showing the expression distribution of the NUMB isoforms and NUMBL in tumour samples.** The upper row shows data from the entire population, while the lower row corresponds to tumour samples paired to non-tumour. The similarity in distribution suggests that the paired subset can be considered a representative group of the entire population.

**Fig S4. Scatter plots comparing Normalized Enrichment Scores (NES) obtained from GSEA analyses between NUMB isoforms and NUMBL.** **A)** The isoform pairs p72/p71, p66/p65, p66/NUMBL, and p65/NUMBL displayed highly similar NES values across all shared GO terms analysed by GSEA. **B)** The isoform pair p72/NUMBL showed completely opposite NES values, suggesting distinct gene set associations for each isoform. A similar pattern was observed for p71/NUMBL and p72/p65, although some GO terms still showed concordant positive NES values. **C)** The isoform pairs p71/p66, p71/p65, and p72/p66 exhibited a more mixed pattern, suggesting partial convergence in pathway associations among these isoforms.

**Fig S5. GSEA analysis shows functional divergence between NUMB isoforms in breast cancer. A)** Comparison of NES values for shared KEGG terms across NUMB isoform groups. Each line connects the average NES value for the same KEGG term between the p72/p71 and p66/p65/NUMBL isoforms. A systematic reversal of the enrichment direction (from negative to positive) is observed, indicating opposing regulation of key biological pathways depending on the expressed isoform. **B)** The opposing association of the citrate cycle (hsa00020) with NUMB isoforms, shown by a negative enrichment trend for p72 and p71 and a positive association with NUMBL, suggests a lower dependence on mitochondrial metabolism when p72/p71 isoforms are predominantly expressed. **C)** The cytokine-cytokine receptor interaction pathway (hsa04060) shows negative enrichment in p72/p71-associated profiles and positive enrichment in p65/NUMBL-associated profiles, indicating an opposite association with this immune pathway depending on the predominant isoform. **D)** The hormonal signalling pathway (hsa04081) is negatively associated with p72/p71 and positively with p65/NUMBL, reflecting an inverse correlation with the expression of these isoforms in relation to hormonal processes. **E)** The Th1 and Th2 differentiation pathway (hsa04658) is exclusively enriched in profiles correlated with p65 and NUMBL, suggesting a specific link with active immune functions. **F)** The AMPK signalling pathway (hsa04152), typically associated with tumour suppressor functions, shows negative enrichment only in association with the p72 and p71 isoforms. RES: Running Enrichment Score; RLM: Rank List Metric.

**Fig S6. Dynamic transcriptional remodelling of key signalling pathways in PDX tumours. A)** Cumulative log2FC in gene expression for multiple biological pathways across serial PDX passages compared to Resection. After implantation in mice, most signalling pathways show a marked downregulation, whereas pathways such as OXPHOS and cell cycle genes increase progressively with each passage, indicating a shift in tumour transcriptional programs. **B)** Gene-level expression changes for individual isoforms in representative signalling pathways (Notch, SHH, WNT, and Hippo). For each pathway, isoform expression is compared between P0 vs Resection (left plots) and P4 vs P0 (right plots). After implantation (P0), a general suppression of key genes is observed (blue dots), indicating early transcriptional reprogramming upon xenografting. Over subsequent passages (P4), partial recovery of gene expression is evident (red dots), suggesting progressive reactivation of these signalling pathways in the evolving PDX environment.

**Fig S7. Isoform-level survival analyses and NUMB-score distribution in the TCGA-BRCA cohort. A)** Kaplan–Meier overall survival curves for individual NUMB isoforms (p72, p71, p66, p65) and NUMBL in the TCGA-BRCA cohort, using scan-derived cutpoints with a minimum group size constraint. p66 and NUMBL show significant associations with survival, whereas p72, p71, and p65 do not. Numbers at risk are shown below each plot. **B)** Density distribution of NUMB-score values in tumour and non-tumour samples from TCGA-BRCA. The dashed line indicates the cut-off used to stratify samples. **C)** Kaplan–Meier overall survival analysis based on NUMB-score stratification in the full TCGA-BRCA cohort (without removal of influential samples), showing reduced survival in the high NUMB-score group. Hazard ratio (HR), 95% confidence interval (CI), and log-rank p-value are indicated.
