# Supplementary material for: Tumoral switch in NUMB splicing changes essential transcription pathways and induces malignant properties in tumour cells
Source: Genome Biol. 2026 Jun 24;27:285. doi: 10.1186/s13059-026-04170-3 (PMC13551979; doi:10.1186/s13059-026-04170-3)
Supplement: Supplementary file 2 [file 13059_2026_4170_MOESM2_ESM.docx]

| **Table S1**. Abbreviations used in **Figure 1**. | | |
| --- | --- | --- |
| Category | Abbreviation | Full name |
| TCGA tumour type | ACC | Adrenocortical carcinoma |
|  | BLCA | Bladder urothelial carcinoma |
|  | BRCA | Breast invasive carcinoma |
|  | CESC | Cervical squamous cell carcinoma and endocervical adenocarcinoma |
|  | CHOL | Cholangiocarcinoma |
|  | COAD | Colon adenocarcinoma |
|  | DLBC | Diffuse large B-cell lymphoma |
|  | ESCA | Esophageal carcinoma |
|  | GBM | Glioblastoma multiforme |
|  | HNSC | Head and neck squamous cell carcinoma |
|  | KICH | Kidney chromophobe |
|  | KIRC | Kidney renal clear cell carcinoma |
|  | KIRP | Kidney renal papillary cell carcinoma |
|  | LAML | Acute myeloid leukemia |
|  | LGG | Lower grade glioma |
|  | LIHC | Liver hepatocellular carcinoma |
|  | LUAD | Lung adenocarcinoma |
|  | LUSC | Lung squamous cell carcinoma |
|  | OV | Ovarian serous cystadenocarcinoma |
|  | PAAD | Pancreatic adenocarcinoma |
|  | PCPG | Pheochromocytoma and paraganglioma |
|  | PRAD | Prostate adenocarcinoma |
|  | READ | Rectum adenocarcinoma |
|  | SARC | Sarcoma |
|  | SKCM | Skin cutaneous melanoma |
|  | STAD | Stomach adenocarcinoma |
|  | TGCT | Testicular germ cell tumors |
|  | THCA | Thyroid carcinoma |
|  | THYM | Thymoma |
|  | UCEC | Uterine corpus endometrial carcinoma |
|  | UCS | Uterine carcinosarcoma |
| AS event type | AA | Acceptor site |
|  | AD | Alternative donor site |
|  | AP | Alternative promoter |
|  | AT | Alternative termination site |
|  | ES | Exon skipping |
|  | IR | Intron retention |
|  | MES | Mutually exclusive exons |
